# Molecular Engineering of Porous Fe‐N‐C Catalyst with Sulfur Incorporation for Boosting CO_2_ Reduction and Zn‐CO_2_ Battery

**DOI:** 10.1002/advs.202407063

**Published:** 2024-08-05

**Authors:** Jingwei Han, Qiang Xu, Jiaxin Rong, Xue Zhao, Ping She, Jun‐Sheng Qin, Heng Rao

**Affiliations:** ^1^ State Key Laboratory of Inorganic Synthesis and Preparative Chemistry College of Chemistry International Center of Future Science Jilin University Changchun Jilin 130012 P. R. China

**Keywords:** coordination environment, electrochemical CO_2_ reduction, molecular engineering, single‐atom catalysts, Zn‐CO_2_ batteries

## Abstract

Transition metal‐nitrogen‐carbon (M‐N‐C) catalysts have emerged as promising candidates for electrocatalytic CO_2_ reduction reaction (CO_2_RR) due to their uniform active sites and high atomic utilization rate. However, poor efficiency at low overpotentials and unclear reaction mechanisms limit the application of M‐N‐C catalysts. In this study, Fe‐N‐C catalysts are developed by incorporating S atoms onto ordered hierarchical porous carbon substrates with a molecular iron thiophenoporphyrin. The well‐prepared FeSNC catalyst exhibits superior CO_2_RR activity and stability, attributes to an optimized electronic environment, and enhances the adsorption of reaction intermediates. It displays the highest CO selectivity of 94.0% at −0.58 V (versus the reversible hydrogen electrode (RHE)) and achieves the highest partial current density of 13.64 mA cm^−2^ at −0.88 V. Furthermore, when employed as the cathode in a Zn‐CO_2_ battery, FeSNC achieves a high‐power density of 1.19 mW cm^−2^ and stable charge–discharge cycles. Density functional theory calculations demonstrate that the incorporation of S atoms into the hierarchical porous carbon substrate led to the iron center becoming more electron‐rich, consequently improving the adsorption of the crucial reaction intermediate *COOH. This study underscores the significance of hierarchical porous structures and heteroatom doping for advancing electrocatalytic CO_2_RR and energy storage technologies.

## Graphical abstract

By utilizing a strategic combination of molecular metalloporphyrins (with a clear and modifiable structure) and heteroatom doping (enabling control over the microenvironment of active sites), atomically dispersed iron species confined within channel‐rich S‐doped nitrogen–carbon materials are synthesized. Electrochemical assessments and theoretical calculations indicate that FeSNC exhibits significant activity and selectivity in CO_2_ reduction reactions.

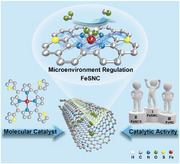

## 1 Introduction

Single‐atom catalysts (SACs) with flexible and controllable active sites and an ultra‐high atomic utilization rate have attracted considerable attention in energy storage and conversion applications.^[^ ^1^, ^2^, ^3^ ^]^ Metal‐nitrogen‐carbon (M‐N‐C) SACs have demonstrated remarkable performance in CO_2_ reduction reaction (CO_2_RR) due to their distinctive chemical structure.^[^ ^4^, ^5^, ^6^ ^]^ Specifically, the exceptional catalytic performance is attributed to the favorable interaction between the atomic metal and the carbon‐oxygen intermediate, facilitated by the unoccupied *d* orbitals of the central transition metal atom.^[^ ^7^ ^]^ Furthermore, adjusting the composition of the conductive carbon skeleton positively influences the regulation of charge distribution at the metal center and electron conduction during the catalytic process.^[^ ^8^ ^]^ Thus, the design and development of cost‐effective M‐N‐C SACs (M = transition metal) with high activity, selectivity, and stability are essential for advancing research on electrocatalytic CO_2_RR. High active site density and strong charge‐ and mass‐transport capabilities are essential for boosting the catalytic performance of catalysts.^[^ ^9^ ^]^ Compared to bulk M‐N‐C materials, M‐N‐C catalysts with porous structures offer various advantages, such as larger specific surface area and more active sites.^[^ ^10^, ^11^, ^12^, ^13^ ^]^ The porous architecture prevents material from aggregation during pyrolysis.^[^ ^14^, ^15^ ^]^ The abundant pore network facilitates reactant molecules to access both the internal and exterior spaces of the catalyst, acting as a molecular reactor and promoting reactant enrichment.^[^ ^16^, ^17^, ^18^ ^]^ Furthermore, the pore structure as a molecular channel greatly improves the diffusion of reactants and products.^[^ ^19^, ^20^, ^21^ ^]^

Doping heteroatoms to optimize the microenvironment of the central metal atom is another proven method for enhancing the intrinsic activity of M‐N‐C catalysts.^[^ ^22^, ^23^, ^24^, ^25^ ^]^ Sulfur atoms can delicately modify the electronic environment of metal active sites due to their greater atomic radius and lower electronegativity compared to nitrogen atoms.^[^ ^26^, ^27^, ^28^ ^]^ The sulfur‐donated electronic contribution greatly enhances electrocatalytic activity. Furthermore, previous research has demonstrated that the microenvironment of secondary coordination spheres plays a crucial role in enhancing the selective binding of substrates to metal centers. By controlling the structure of the second coordination sphere, it is possible to stabilize high‐energy intermediates or transition states, facilitate the selective transfer of protons to the bound substrate, and even modify the oxidation state of the metal species. These modifications influence kinetics, thermodynamics, and reaction mechanisms to enhance catalytic efficiency.^[^ ^29^, ^30^, ^31^, ^32^, ^33^, ^34^ ^]^ For instance, Pan et al. synthesized an N and S dual‐doped Fe‐NS‐C catalyst with microporous carbon layers using a copolymer‐assisted approach to facilitate the CO_2_RR. The first‐principles calculations demonstrate that the improved activity of Fe‐NS‐C is attributed to incorporating S atoms, leading to increased charge density on Fe atoms of Fe‐N_4_ and a reinforced interaction between the Fe site and the crucial COOH* intermediate.^[^ ^35^ ^]^ Similarly, Tang et al. doped S into the second shell of the nickel site to synthesize a Ni‐N_4_‐SC catalyst that exhibited outstanding performance and durability in CO_2_RR. The strategical placement of S atoms in the second shell reduced the energy barrier of the CO_2_RR. It also counteracted the challenges posed by the larger atomic radius of S, ultimately improving the overall durability of the catalyst.^[^ ^36^ ^]^ These studies provide strong evidence that incorporating S atoms into the carbon structure effectively modifies the local electron density and enhances the adsorption of intermediates. However, most literature reported that S‐doped M‐N‐C catalysts were often synthesized from two or three separate precursors, serving as the metal source, C/N source, and S source, respectively.^[^ ^37^, ^38^, ^39^, ^40^ ^]^ This variety of precursor sources complicates the determination of the ultimate catalyst structure, presenting a notable obstacle to comprehending the catalytic mechanism. Therefore, it is essential to identify appropriate precursors when synthesizing well‐defined heteroatom‐doped M‐N‐C catalysts. These precursors should possess a clear structure and facilitate the integration of heteroatoms, C/N atoms, and metal atoms within the same material. Metalloporphyrin molecules containing heteroatoms are considered to be one of the most optimal choices for the accurate construction of heteroatom‐doped M‐N‐C catalysts due to their significant advantages in regulating structure and composition.^[^ ^41^ ^]^ Upon pyrolysis, these molecules are transformed into nitrogen‐doped carbon frameworks. The nitrogen atoms in these molecules coordinate with metal atoms in the form of pyridine or pyrrole N. Moreover, the abundance of heteroatoms in porphyrin molecules facilitates the incorporation of heteroatom doping into the carbon matrix. It is crucial to note that porphyrin molecules containing heteroatoms possess a unique structure that integrates carbon, nitrogen, metals, and heteroatoms within a single unit, making them an ideal platform for the precise construction of heteroatom‐doped M‐N‐C catalysts.

This study demonstrates the synthesis of atomically dispersed iron species confined within channel‐rich S‐doped nitrogen‐carbon materials using a carefully designed pyrolysis strategy, which enhances the CO_2_RR efficiency. The scheme begins with a targeted molecular design, focusing on incorporating S atoms in situ and constructing precise catalytic architectures. Iron thiophenoporphyrin molecules are initially prepared to serve as both the precursor of an N‐doped carbon skeleton and as a source of Fe and S for subsequent processes. These molecules are confined in SBA‐15 channels and polymerized to create an ordered framework that helps prevent metal atom aggregation. Subsequently, pyrolysis is carried out to produce a porous FeSNC catalyst. The porous structure facilitates the mass transfer and exposure of active sites during catalysis. With this unique in situ doping approach, the original Fe‐N_4_ active sites are preserved while the S atoms from the iron thiophenoporphyrin molecules are incorporated into the N‐doped carbon framework. These S atoms are precisely positioned in the second shell of the Fe‐N_4_ active site to optimize the electronic microenvironment. Electrochemical tests demonstrate that FeSNC exhibits high CO_2_RR activity and selectivity. Theoretical calculations reveal that S doping in the second shell optimizes the electronic structure of the Fe single‐atom site, improving the adsorption of the critical reaction intermediate *COOH, thereby boosting CO_2_ electroreduction activity and stability. The heteroatom‐containing porphyrin‐derived M‐N‐C catalysts, which combine molecular metal‐porphyrin (clear and tunable structure) and heteroatom doping (controlling the microenvironment of the active site), provide valuable insights for the precise design of heteroatom‐doped M‐N‐C catalysts with well‐defined structures.

## 2 Results and Discussion

**Figure 1:**
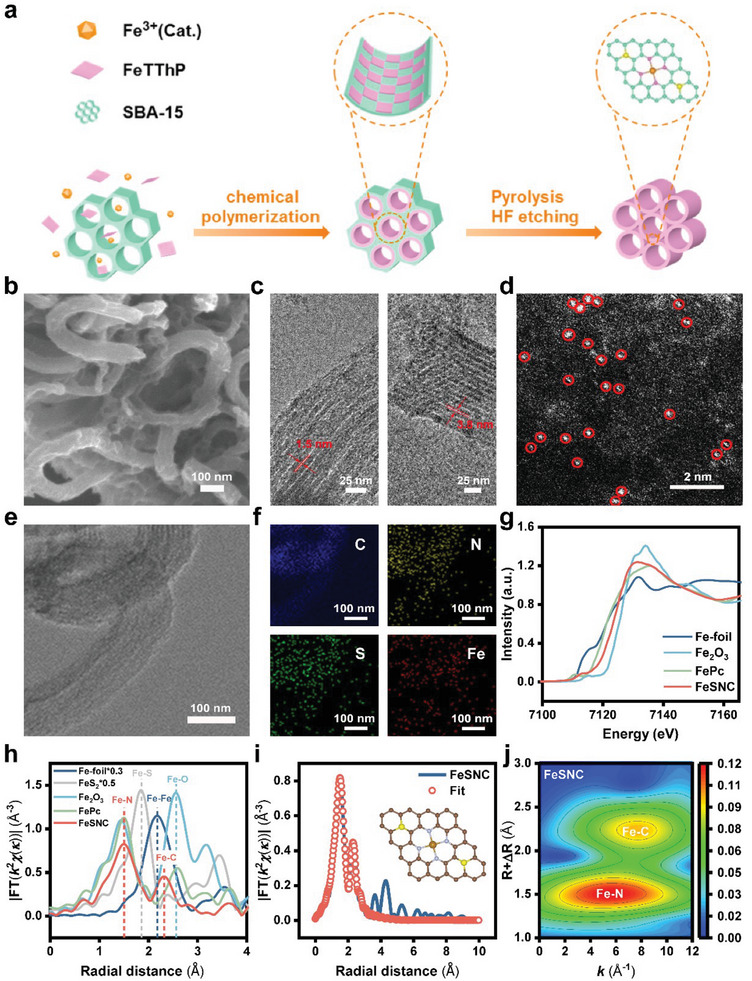
a) Schematic illustration of the synthesis of FeSNC. b) SEM image, c) TEM images, d) AC‐HAADF‐STEM image of FeSNC. e,f) Elemental mapping of FeSNC reveals uniform distributions of C (blue), N (yellow), S (green), and Fe (red). g) Fe K‐edge XANES spectra and h) Fourier transformation EXAFS spectra of FeSNC and reference samples. i) The fitting EXAFS spectra of FeSNC and fitting model. j) WT k^2^‐weighted EXAFS contour plot of the FeSNC.

Free‐base tetraphenylporphyrin (H_2_TPP = tetra(*meso*‐phenyl)porphyrin) and thiophenoporphyrin (H_2_TThP = tetra(*meso*‐thien‐2‐yl)porphyrin) were synthesized using the Adler method, as detailed in the Supplementary Information. ^1^H nuclear magnetic resonance (^1^H NMR) spectroscopy proved the synthesis of H_2_TPP and H_2_TThP (Figure S1, Supporting Information). Following metallization in the presence of FeCl_2_·4H_2_O yields iron tetraphenylporphyrin (FeTPP = iron(III) tetra(*meso*‐phenyl)porphyrin chloride) and iron thiophenoporphyrin (FeTThP = iron(III) tetra(*meso*‐thien‐2‐yl)porphyrin chloride). They were characterized using UV–vis spectroscopy, mass spectrometry, and cyclic voltammetry (Figures S2–S4, Supporting Information). Subsequently, SBA‐15 was incorporated into a chloroform solution containing FeTThP and FeCl_3_, and subjected to negative pressure for 2 h to facilitate the penetration of FeTThP into the pores of SBA‐15. It is noteworthy that FeCl_3_ served as a catalyst in this process to facilitate the polymerization of FeTThP, rather than being utilized as a source of iron. Finally, the FeSNC with hierarchical pores was obtained through pyrolysis and etching (**Figure** **1**a).

The scanning electron microscope (SEM) images of the prepared FeSNC reveal that it maintains the typical rod‐like structure of the SBA‐15 molecular sieve (Figure 1b). Additionally, transmission electron microscope (TEM) images (Figure 1c) depict the well‐ordered micro‐mesoporous structure of FeSNC, which is consistent with previous literature.^[^ ^42^ ^]^ In the aberration‐corrected high‐angle annular dark‐field scanning transmission electron microscopy (AC‐HAADF‐STEM) image of FeSNC (Figure 1d), distinct and isolated dense bright spots are observed scattered across the carbon matrix, confirming the presence of Fe elements distributed as single atoms. The elemental mapping of FeSNC confirms that C, N, S, and Fe are evenly distributed in the matrix (Figure 1e,f).

The coordination structure of Fe atoms in FeSNC was investigated using X‐ray absorption spectroscopy (XAS). For comparison, Fe foil, commercial iron phthalocyanine (FePc), Fe_2_O_3_, and FeS_2_ are used as reference samples. The absorption edge position of FeSNC is observed in the X‐ray absorption near edge structure (XANES) of the Fe spectrum (Figure 1g). Notably, the absorption edge position of FeSNC closely resembles that of FePc. In Figure 1h and Figure S5 (Supporting Information), the Fourier‐transformed (FT) k^2^‐weighted extended X‐ray absorption fine structure (EXAFS) spectra of Fe foil and FePc exhibit two different peaks located at 2.18 and 1.50 Å, respectively. The main peaks of FeSNC are observed at 1.50 and 2.33 Å corresponding to Fe‐N coordination and Fe‐C coordination, respectively.^[^ ^43^, ^44^, ^45^ ^]^ No Fe‐Fe bonding signal is observed indicating that the as‐prepared FeSNC in this work is a single‐atom catalyst. Fe‐O coordination and Fe‐S coordination were not observed when compared with Fe_2_O_3_ and FeS_2_. As shown in Figures 1i, Figure S6, and Table S1 (Supporting Information), the fitting results confirm that each Fe atom in FeSNC is coordinated with four N atoms. The wavelet transform (WT) EXAFS (Figure 1j; Figure S7, Supporting Information) supported the above results. S atoms are not directly coordinated with iron but are embedded in the carbon substrate to form a unique structure. These S atoms, positioned in the second shell, play a dual role in stabilizing Fe‐N_4_ and modulating the charge distribution of the central iron atom.^[^ ^36^ ^]^

X‐ray photoelectron spectroscopy (XPS) was employed to analyze the elemental composition and coordination environment. The S 2p spectrum of the FeSNC (Figure S8, Supporting Information) displays peaks at 163.7 eV and 165.0 eV, which correspond to the 2p_3/2_ and 2p_1/2_ branches of the C‐S‐C (N) species.^[^ ^28^ ^]^ The peaks at 167.9 eV and 169.2 eV are attributed to the oxidized S moiety (C‐SO_x_‐C).^[^ ^28^ ^]^ The XPS analysis results align well with those obtained from XAS. The FeSNC catalyst exhibited a sulfur concentration twice as high as that of the reported reference (Table S2, Supporting Information).^[^ ^28^ ^]^ This indicates that directly doping sulfur atoms into the carbon matrix from the thiophenoporphyrin is a more efficient method than doping with a sulfur source alone. Therefore, selecting the appropriate S source for doping is crucial for controlling the content and structure of the catalyst.

For making FeSNC, FeTThP was utilized as both a sulfur source and a precursor. For comparison, the FeNC was prepared based on the same method using FeTPP instead of FeTThP. While in the preparation of FeNCS, thiourea was employed as a sulfur source alongside FeTPP as a precursor. A non‐porous catalyst, pFeTThP, was synthesized by pyrolyzing FeTThP without SBA‐15 to assess the effect of hierarchical pore structure (Figure S9, Supporting Information). Morphological analysis (Figures S10 and S11, Supporting Information) reveals that FeNC and FeNCS exhibit similar pore structures to FeSNC, while pFeTThP lacks any discernible pores.

**Figure 2:**
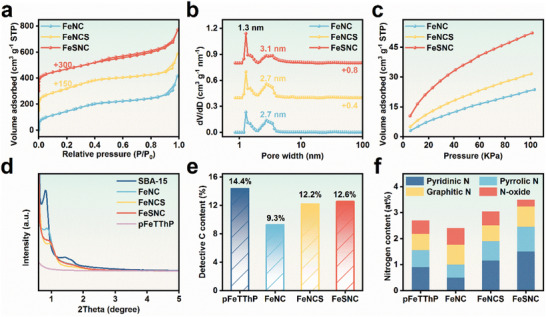
a) N_2_ adsorption‐desorption isotherms at 77 K, b) pore size distribution, and c) CO_2_ adsorption isotherms at 273 K for FeNC, FeNCS, and FeSNC. d) Small angle XRD of SBA‐15 and prepared catalysts. e) Content of detective C derived from the C 1s XPS analysis. f) Content of nitrogen species derived from the N 1s XPS analysis.

The specific surface area and pore structure of three porous materials were determined using the N_2_ adsorption‐desorption method. The N_2_ adsorption‐desorption isotherms for the three materials (**Figure** **2**a) indicate similar structures. The Brunauer‐Emmett‐Teller (BET)‐specific surface areas of FeNC, FeNCS, and FeSNC are calculated to be 553.52, 639.66, and 605.52 m^2^ g^−1^, respectively (Table S3, Supporting Information). The larger specific surface area of the material suggests S doping results in more defects and active sites.^[^ ^46^ ^]^ The pore distribution curve, obtained using the density functional theory (DFT) method, is shown in Figure 2b. All catalysts manifest a hierarchical porous structure, consisting of micropores and mesopores. This unique structure is a result of the two‐pore sizes present in the SBA‐15 raw material, leading to the formation of an ordered hierarchical pore carbon material during the pyrolysis process (Figure S12, Supporting Information). Among the three porous materials, FeSNC has the largest pore volume of 0.73 cm^3^ g^−1^ (Table S3, Supporting Information). The hierarchical pore structure has been approved to enhance mass transport.^[^ ^42^ ^]^ Furthermore, both FeNCS and FeSNC possess greater CO_2_ affinity than FeNC (Figure 2c).

The pore structure of the synthesized catalysts was also analyzed using small‐angle X‐ray scattering (SAXS) and compared with SBA‐15. Figure 2d shows that SBA‐15 exhibits scattering peaks with higher resolution in the low‐angle region, indexed to (100) and (200).^[^ ^47^ ^]^ For FeNC, FeNCS, and FeSNC catalysts, a distinct (100) peak was still observed, with slightly reduced intensity, indicating that the ordered mesoporous structure was still maintained even after the SBA‐15 skeleton was etched. The pFeTThP catalyst, on the other hand, did not incorporate the SBA‐15 template during the synthesis process and consequently lacked any discernible pore structure in its final form. Furthermore, as depicted in Figure S13 (Supporting Information), the powder X‐ray diffraction (XRD) patterns reveal that the diffraction patterns of FeNC, FeNCS, and FeSNC are quite similar. The significant peaks at 24.8° and 43.8° represent the (002) and (100) planes of carbon, respectively.^[^ ^42^ ^]^ Notably, no distinct peaks corresponding to Fe‐based nanoparticles were observed in the XRD pattern, suggesting the absence of such nanoparticles in the catalyst. These findings are in accordance with the previous results obtained from TEM and HAADF‐STEM.

The iron contents of pFeTThP, FeNC, FeNCS, and FeSNC were measured by the inductively coupled plasma optical emission spectrometry (ICP‐OES) and found to be 0.06, 0.20, 0.17, and 0.13 wt%, respectively (Figure S14, Supporting Information). Due to the limited Fe content in the catalyst, further analysis of the Fe 2p spectra of all samples is challenging (Figures S15 and S16, Supporting Information). However, the results mentioned above also indicate that there are no Fe‐based nanoparticles present on the material's surface. The C 1s spectrum exhibits three fitting peaks (Figure S17, Supporting Information), which correspond to C═C (284.6 eV), C─N/C─S (285.7 eV), and O─C═O (288.8 eV).^[^ ^40^, ^48^ ^]^ According to previous literature, the peak assigned to C‐N/C‐S represents defective carbon.^[^ ^40^ ^]^ The defective carbon content in FeSNC is 3.3% higher than in FeNC, while in FeNCS it is 2.9% higher than in FeNC (Figure 2e). A similar conclusion was reached by the Raman results (Figure S18, Supporting Information). Figure S19 (Supporting Information) manifests that the peaks at 399.6, 400.8, 402.2, and 405.2 eV in the N 1s spectrum represent pyridinic N coordinates with metals, un‐coordinating pyrrolic N, graphitic N, and N‐oxide, respectively.^[^ ^49^, ^50^, ^51^ ^]^ The distribution of different N species in these catalysts is illustrated in Figure 2f and Table S2 (Supporting Information). Pyridinic content appears to rise with S doping. This is a beneficial phenomenon, as pyridinic N contributes to the stability of iron atoms by coordinating with them.^[^ ^40^, ^52^ ^]^ Similarly, the XPS results indicate that Fe─S bonds are absent in the FeNCS and pFeTThP samples. (Figure S20, Supporting Information).

**Figure 3:**
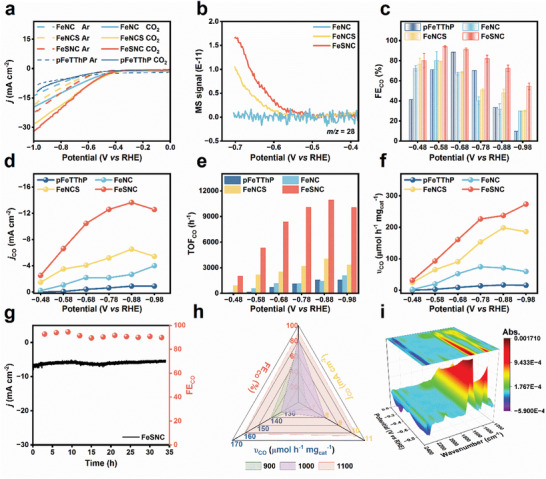
a) The LSV curves of FeNC, FeNCS, FeSNC, and pFeTThP samples in Ar and CO_2_ saturated 0.5 m KHCO_3_. b) The signal of *m/z* = 28 changes with potential in the DEMS test of FeNC, FeNCS, and FeSNC. c) FE_CO_, d) partial *j*_CO_, e) TOF_CO_, and f) the CO productivity variation of FeNC, FeNCS, FeSNC, and pFeTThP samples. g) Stability test and FEs of CO product for the FeSNC. h) FE_CO_, *j*_CO,_ and the CO productivity variation of FeSNC‐T (T refers to the temperature of pyrolysis) at −0.68 V versus RHE. i) In situ ATR‐SEIRAS spectroscopy of FeSNC at different applied potentials.

The CO_2_RR performance of these catalysts was studied in an H‐type cell with a three‐electrode system. The two chambers are separated by a proton exchange membrane, and both contain 30 mL of 0.5 M KHCO_3_ electrolyte. Before the bulk electrolysis, CO_2_ gas was bubbled in the electrolyte for 30 min to remove dissolved oxygen. In linear sweep voltammetry (LSV) curves (**Figure** **3**a), FeSNC demonstrates a higher overall current density (*j*） and a lower onset potential (−0.40 V versus RHE) compared to the other catalysts. This finding is further supported by the results of differential electrochemical mass spectrometry (DEMS) (Figure 3b). The onset potential measured by DEMS appears to be more negative than that measured by the electrochemical workstation, which is attributed to the hysteresis of gas absorption by the instrument.^[^ ^53^ ^]^ Gas chromatography detected only two gas phase products, CO and H_2_. The electrolyte was tested using ion chromatography (IC) to confirm the absence of formic acid and acetic acid. ^1^H NMR spectra showed no other liquid products. Controlled potential electrolysis (Figure S21, Supporting Information) was conducted to investigate the Faradaic efficiency (FE) and catalytic activity of the catalysts. According to Figure 3c, FeSNC demonstrates the highest FE_CO_ of all the catalysts evaluated. FeSNC achieves a maximum FE_CO_ of 94.0% at −0.58 V versus RHE, while FeNC, FeNCS, and pFeTThP manifest FE_CO_ values of 80.2%, 79.3%, and 71.0% at −0.58 V versus RHE, respectively. Across all investigated potentials, FeSNC displays a higher *j*_CO_ than the other catalysts (Figure 3d). The turnover frequency (TOF) was calculated based on the amount of Fe modification on the electrode surface. Similarly, FeSNC exhibits a higher TOF_CO_ at all electrolysis potentials (Figure 3e). The highest TOF_CO_ of 10, 933 h^−1^ is achieved at −0.88 V versus RHE, which is 7, 8, and 3 times higher than that of pFeTThP, FeNC, and FeNCS, respectively. In Figure 3f, the productivity variation of CO for FeSNC remained consistently high, reaching a peak of 273.55 µmol h^−1^ mg_cat_^−1^. Notably, the *j*_CO_, FE_CO_, and productivity variation of CO for FeNC with a pore structure were all higher than those for pFeTThP. The research findings indicate that the presence of hierarchical pores accelerates mass transfer, leading to boosted catalytic activity. Moreover, the catalytic activity of FeSNC and FeNCS was further enhanced through S doping, particularly with in situ S doping showing a greater positive impact. Overall, the exceptional selectivity and activity of FeSNC for CO_2_RR is attributed to the combined effects of the hierarchical pore structure and in situ S doping. To evaluate the durability of the catalyst, electrolysis was performed for 34 h at a constant potential of −0.58 V. The results are illustrated in Figure 3g and Figure S22 (Supporting Information). Following a 34‐h electrolysis, the current densities of the three catalysts with porous structures (FeNC, FeNCS, and FeSNC) all presented a certain degree of decline, with reductions of 45%, 24%, and 7%, respectively. This suggests that FeSNC demonstrates impressive stability.

The impact of pyrolysis temperature on catalyst activity has also been investigated, revealing that FeSNC‐1100 pyrolyzed at 1100 °C exhibited the highest performance (Figure 3h). The *j*_CO_ of FeSNC‐1100 was superior to that of FeSNC‐900 and FeSNC‐1000, possibly attributed to the enhanced graphitization of FeSNC‐1100 (Figure S23, Supporting Information). The increased graphitization improves the catalyst's conductivity (Figure S24, Supporting Information), resulting in a higher current density. Furthermore, properties such as iron content and pore structure were also analyzed in the FeNSC‐T catalyst to further elucidate the reasons behind the excellent catalytic performance of FeSNC‐1100. Overall, FeSNC‐1100 demonstrates superior catalytic performance compared to the other catalysts, influenced by factors such as iron content, pore structure, and conductivity (Figure S25 and Tables S4 and S5, Supporting Information).

To investigate the reasons for the high performance of FeSNC, the electrochemically active surface area (ECSA) was estimated by measuring the double‐layer capacitance (C_dl_), as illustrated in Figure S26 (Supporting Information). Surprisingly, the ECSA values of FeNC (25.19 mF cm^−2^), FeNCS (28.11 mF cm^−2^), and FeSNC (24.38 mF cm^−2^) did not exhibit a direct correlation with their selectivity and activity. This indicates that the activity and selectivity of CO_2_RR are not influenced by the specific surface area. Subsequently, electrochemical impedance tests were conducted to analyze the electron transfer kinetics in CO_2_RR (Figure S27, Supporting Information). The charge transfer resistances (R_ct_) of the three catalysts were observed to be minimal, signifying their excellent conductivity. Notably, FeSNC displays the smallest R_ct_, implying the least resistance to charge transfer during the CO_2_RR. In situ attenuated total reflection surface‐enhanced infrared absorption spectroscopy (ATR‐SEIRAS) was employed to investigate the CO_2_RR process. A negative peak at 2334 cm^−1^ was detected, indicating the consumption of CO_2_.^[^ ^54^ ^]^ As the CO_2_RR advanced toward a more negative potential, a characteristic CO peak at 2057 cm^−1^ was observed for FeSNC (Figure 3i; Figure S28, Supporting Information).^[^ ^40^ ^]^ As the potential decreased, the intensity of the absorption peaks located at 1395 cm^−1^ (*COOH/HCO_3_^−^) and 1650 cm^−1^ (H─O─H) increased. This phenomenon is closely associated with the orderliness of the interfacial water structure.^[^ ^55^ ^]^ The same intermediates in the CO_2_RR process are also seen for FeNC and FeNCS, implying their reaction paths are similar.

The efficacy of the catalyst in the oxygen evolution reaction (OER) was assessed by recording LSV curves in a 0.5 M KHCO_3_ electrolyte. Figure S29 (Supporting Information) illustrates that the current density remains stable within the potential range of 1.40–1.80 V versus RHE. As the potential increases, the current density gradually rises and sharply increases after reaching 2.20 V versus RHE, corresponding to the OER. The FeSNC catalyst exhibits an initial overpotential of 0.52 V. When compared to other catalysts (FeNC, FeNCS, and pFeTThP), the FeSNC catalyst demonstrates a lower overpotential of 1.08 V at a current density of 20 mA cm^−2^. Analysis of the OER kinetics on the catalyst reveals that FeSNC exhibits the lowest Tafel slope (Figure S30, Supporting Information).

**Figure 4:**
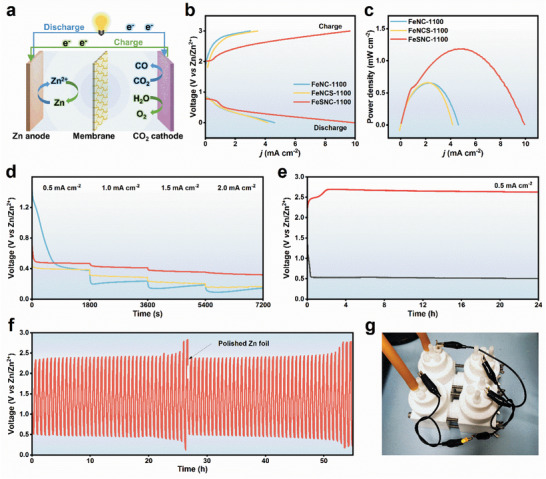
a) Schematic configuration of the Zn‐CO_2_ battery. b) Discharge and charge polarization curves at a scanning rate of 5 mV s^−1^. c) Power density curves of catalysts. d) Discharge voltages at different currents. e) Prolonged galvanostatic charge and discharge curves at 0.5 mA cm^−2^. f) Galvanostatic discharge–charge cycling curves at the current density of 0.5 mA cm^−2^. g) Digital photograph of an LED illuminated by two Zn‐CO_2_ batteries in series.

Based on the bifunctional CO_2_RR/OER activities of the catalyst, a battery setup was devised with coated carbon paper as the positive electrode and zinc sheets as the negative electrode (**Figure** **4**a). The charge–discharge polarization curves and corresponding power densities of the Zn‐CO_2_ battery are illustrated in Figure 4b,c. FeSNC demonstrates a current density of 10 mA cm^−2^ at a discharge voltage of 0 V and a charge voltage of 3 V. It achieves a maximum power density of 1.19 mW cm^−2^ at a current density of 4.6 mA cm^−2^, outperforming other catalysts and recently reported catalysts (Table S6, Supporting Information). During discharge at a constant current density of 0.5–2.0 mA cm^−2^, the discharge voltage of FeSNC consistently surpassed that of FeNC and FeNCS (Figure 4d). The operational durability of the Zn‐CO_2_ battery with FeSNC cathode was assessed through single charge–discharge tests and multiple charge–discharge cycle tests at 0.5 mA cm^−2^. The battery maintains a stable test curve with a consistent discharge voltage of 0.50 V during a single charge–discharge cycle (Figure 4e). The voltage gap between charge and discharge widens from 1.84 to 2.73 V after 38 cycles. After polishing and reassembling the Zn foil, the voltage gap reduces to 1.94 V. Unfortunately, this gap increases again after 40 cycles due to ZnO deposition on the membrane and precipitation in the anode chamber (Figure 4f).^[^ ^56^ ^]^ In Figure 4g, two Zn‐CO_2_ batteries with FeSNC cathodes connected in series light up a yellow light‐emitting diode. This study validates the FeSNC catalyst as a reversible Zn‐CO_2_ battery cathode material with efficient energy conversion and output capabilities.

**Figure 5:**
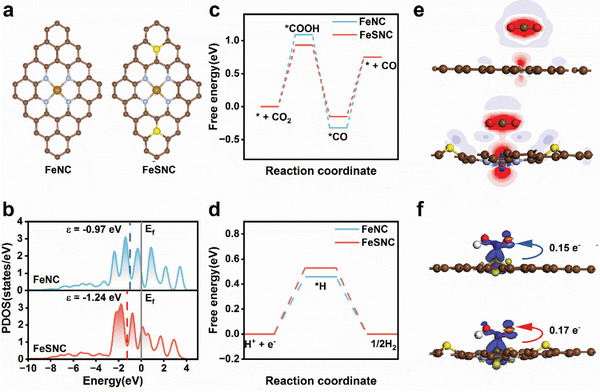
a) Theoretically. Computed model of FeNC (left) and FeSNC (right). (C: brown, N: blue, Fe: gold, S: yellow) b) The projected density of states (PDOS) of d orbitals of Fe atoms on FeNC and FeSNC catalysts. The grey lines represent the Fermi level, and the dashed lines denote the d‐band centers (ε). Free‐energy paths of intermediates in c) CO_2_RR and d) HER on FeNC and FeSNC. e) The valence charge density difference (VCDD) maps for CO_2_ adsorption on FeNC (upper) and FeSNC (lower). Red and blue isosurfaces show electron accumulation and depletion, respectively. f) Differential charge densities of FeNC (upper) and FeSNC (lower) after the *COOH intermediate was adsorbed near the Fe atom. isosurface = 0.02 e/Å^3^. Blue and yellow isosurfaces show the electron increase and decrease, respectively. (C: brown, N: blue, Fe: gold S: yellow, O: red, H: white).

DFT calculations were performed to further understand the enhanced reaction kinetics of FeSNC. A Fe‐N_4_ model (FeNC) and a Fe‐N_4_‐SC model with second shell S‐doping (FeSNC) were constructed predicated on EXAFS and XPS results (**Figures** **5**a and S31, Supporting Information). The S dopant was strategically built further away from the Fe atoms to better illustrate the S doping effect, given that thiophene‐S was located at the edges of Fe porphyrin. A negative shift in the d band center of Fe atoms on FeSNC (−1.24 eV) compared to FeNC (−0.97 eV) was found by examining the projected density of states (PDOS) and band centers (ε) of Fe 3d orbitals in the models (Figure 5b). This resulted in a weaker binding to reaction intermediates such as *COOH and *CO.^[^ ^26^ ^]^ This weaker binding was expected to enhance the CO_2_RR activity on FeSNC. Moreover, the negative shift of the ε in FeSNC enhanced the Lewis basicity of the Fe center, facilitating the capture of CO_2_ and electron transfer. Based on previous research on similar M‐N‐C catalysts and their reaction mechanisms,^[^ ^6^, ^50^ ^]^ Figures 5c and S32 (Supporting Information) depict the free energy diagram for the reduction of CO_2_ to CO via *COOH and *CO intermediates on FeNC and FeSNC. The rate‐limiting steps of both FeNC and FeSNC involved * + CO_2_ + H^+^ + e^−^→ *COOH, with *ΔG* values of 1.09 and 0.93 eV, respectively. This implies that FeSNC exhibits superior catalytic activity. An effective CO_2_RR catalyst should exhibit low activity toward the HER, as it is a common side reaction in CO_2_RR. Figure 5d presents the calculated free energy diagrams for the HER on FeNC and FeSNC, with FeSNC showing weaker *H adsorption and consequently poor HER activity. Previous research^[^ ^57^, ^58^ ^]^ has highlighted the difference in the limiting potential between CO_2_RR and HER, which was used to indicate the selectivity of CO_2_RR (denoted as U_L_(CO_2_)‐U_L_(H_2_), where U_L_ signifies the maximum free energy change (*ΔGmax*) among all elementary steps along the most favorable pathway). The U_L_(CO_2_)‐U_L_(H_2_) value for the FeNC was more negative than that of the FeSNC, indicating that FeSNC exhibits a higher selectivity for CO_2_RR (Figure S33 and Tables S7 and S8, Supporting Information).

The ease of CO_2_ adsorption on the active site is crucial for the CO_2_RR activity of the catalyst. To visualize the electron transfer during CO_2_ adsorption, the valence charge density difference (VCDD) of two configurations was calculated (Figure 5e). The analysis highlights that FeSNC exhibits a more pronounced electron delocalization structure, suggesting the S‐doped Fe‐N_4_ structure facilitates easier CO_2_ adsorption. To further explore the impact of electron density at Fe sites on CO_2_ activation, differential charge densities were analyzed (Figure 5f). The initial charge values for FeNC and FeSNC are 0.57 and 0.52 e^−^, respectively. After *COOH formation on FeNC and FeSNC, the charge values change to 0.42 and 0.35 e^−^, respectively. This results in a greater charge transfer on FeSNC, leading to a smaller *ΔG* value for the rate‐limiting step. These observations are consistent with experimental results demonstrating significantly boosted CO_2_RR performance with S‐doped FeSNC catalysts.

## 3 Conclusion

In summary, a Fe single‐atom electrocatalyst was synthesized by incorporating sulfur into the second shell of Fe sites on channel‐rich porous carbon supports. This catalyst is highly efficient for CO production in CO_2_RR. Experimental results also highlight FeSNC's efficacy as a cathode catalyst in a Zn‐CO_2_ cell, achieving a peak power density of 1.19 mW cm^−2^ with satisfactory stability. DFT calculations revealed that channel‐rich porous carbon materials with S‐doped active sites optimize the adsorption and activation of intermediates by modifying the electronic structure of d‐states. Furthermore, S atoms in the second shell promote electron transfer and lower the energy barriers of CO_2_RR, ultimately enhancing CO_2_ reduction activity and selectivity. This study offers valuable insights into designing heteroatom‐doped single‐atom catalysts, optimizing the local electronic environment of single‐atom active sites, and advancing sustainable energy conversion through efficient electrocatalytic processes.

## Conflict of Interest

The authors declare no conflict of interest.

## Supporting information

Supporting Information

## Data Availability

The data that support the findings of this study are available in the supplementary material of this article.
