# Supplementary material for: Molecular Engineering of Porous Fe‐N‐C Catalyst with Sulfur Incorporation for Boosting CO2 Reduction and Zn‐CO2 Battery
Source: Adv Sci (Weinh). 2024 Aug 5;11(38):2407063. doi: 10.1002/advs.202407063 (PMC11481232; doi:10.1002/advs.202407063)
Supplement: Supplementary file 1 — Supporting Information [file ADVS-11-2407063-s001.docx]

Supporting Information

Molecular Engineering of Porous Fe-N-C Catalyst with Sulfur Incorporation for Boosting CO_2_ Reduction and Zn-CO_2_ Battery

Jingwei Han,^[a]^ Qiang Xu,^[a]^ Jiaxin Rong,^[a]^ Xue Zhao,^[a]^ Ping She,^[a]^ Jun-Sheng Qin^[a]^ and Heng Rao*^[a]^

[a] J. Han, Q. Xu, J. Rong, X. Zhao, P. She, Prof. J. Qin, Prof. H. Rao

State Key Laboratory of Inorganic Synthesis and Preparative Chemistry, College of Chemistry, International Center of Future Science, Jilin University, Changchun, Jilin 130012, P. R. China.
E-mail: rao@jlu.edu.cn

**Table of contents**

1. Experimental section 3

1.1 Chemical and materials 3

1.2 Synthetic Details 3

1.3 Characterizations 4

1.4 Electrochemical Measurements 5

1.5 Methods 6

1.6 DFT calculations 7

2. Supplementary Figures and Tables. 8

3. References 27

1. Experimental section
2. Chemical and materials

Pluronic P123 (Mw=5800) and tetraethylorthosilicate (TEOS, 98%) were purchased from Aladdin. Hydrochloric acid (HCl, 37%), hydrofluoric acid (HF, 40%), glacial acetic acid (CH_3_COOH, 99.8%), and propionic acid (CH_3_CH_2_COOH, 99.5%) were obtained from Sinopharm Chemical Reagent Co., Ltd. Nitrobenzene (C_6_H_5_NO_2_, 99%) 2-thiophenecarbaldehyde (C_5_H_4_OS, 98%) pyrrole (C_4_H_4_NH, 99%) benzaldehyde (C_6_H_5_CHO, 99%), Tetra-n-butylammonium hexafluorophosphate (TBAPF_6_, 98%), and potassium hydrogen carbonate (KHCO_3_, 99%) were purchased from Beijing InnoChem Science & Technology Co., Ltd. Potassium hydroxide (KOH, 95%), and zinc acetate (Zn(AcO)_2_, 99%) were purchased from Macklin. All electrodes were obtained from Gaoss Union (Tianjin).

1. Synthetic Details

**Preparation of SBA-15 Silica.**

Mesoporous SBA-15 silica was prepared based on literature with slight modification^[1]^. In a typical synthesis, 2.0000 g P123 and 4.2500 g TEOS were mixed in 35.00 mL of 1.6 M HCl and stirred at 40 °C for 24 hours. The mixture was then transferred to a 100 mL Teflon-lined autoclave and reacted at 100 °C for 24 hours. After cooling to room temperature, the white solid was obtained by filtration, washed with water and ethanol in sequence, and dried at 80 °C for 24 hours. Subsequently, the solid powder was calcined in a muffle furnace at 550 °C for 5 hours with a heating rate of 1 °C min^-1^ to remove the template agent.

Finally, the as-prepared SBA-15 silica was acidified in 1.0 M HCl solution at 80 °C for 24 hours, then washed until neutral and dried at 80 °C for 24 hours.

**Preparation of Iron(III) tetra(*meso*-phenyl)porphyrin chloride (FeTPP)^[2]^.**

Tetraphenylporphyrin was synthesized according to the literature^[3]^. Briefly, pyrrole and benzaldehyde undergo a condensation reaction in a propionic acid solution to form tetraphenylporphyrin. The crude product was purified by silica gel column chromatography (chloroform : petroleum ether = 2:1). Typically, dissolved H_2_TPP 615.0 mg (1.0 mmol) in 100.00 ml DMF, then added 795.0 mg FeCl_2_·4H_2_O (4.0 mmol). The resulting mixture was refluxed at 150 °C for 6 h. After cooling to room temperature, wash with deionized water several times, filter the precipitate, and vacuum dry at 60 °C for 12 hours to obtain FeTPP.

**Preparation of** **Iron(III) tetra(*meso*-thien-2-yl)porphyrin chloride (FeTThP)^[4]^.**

To a three-necked flask charged with a stir bar add 30.00 ml of glacial acetic acid, 30.00 ml of propionic acid, and 15.00 ml of nitrobenzene, and refluxed at 130 °C for 20 minutes. 2.80 mL (0.03 mol) of 2-thiophenecarbaldehyde was added to 30.00 mL of propionic acid and 2.07 mL (0.03 mol) of pyrrole was added to 30.00 mL of acetic acid. Then, the above solution was slowly dropped into the flask at the same time and refluxed at 130 °C for 50 minutes. After the reaction cooled to room temperature, 25.00 ml of methanol was added and allowed to stand overnight. It was then filtered and washed with hot water and ethanol until the filtrate is colorless. After drying for 24 hours under vacuum, the crude product was purified by silica gel column chromatography (chloroform : petroleum ether = 2:1).

Typically, dissolved H_2_TThP 639.0 mg (1.0 mmol) in 100.00 ml DMF, then added 795.0 mg FeCl_2_·4H_2_O (4.0 mmol). The resulting mixture was refluxed at 150 °C for 6 hours. After cooling to room temperature, wash with deionized water several times, filter the precipitate, and vacuum dry at 60 °C for 12 hours to obtain FeTThP.

**Preparation of FeNC**

SBA-15 silica was employed as a template for the synthesis of highly ordered porous Fe, N-doped carbon. First, 100.0 mg of FeTPP was lysed in 100.00 ml of DMF, and then 100.0 mg of hydrochloric acid-treated SBA-15 was added to the solution. The mixture was kept under sonication and vacuum at 50 °C for 1 hour and then stirred vigorously at 50 °C for 10 hours. DMF was then distilled off under reduced pressure. The obtained composite was transferred to a porcelain boat and pyrolyzed at 1100 °C with a heating ramp rate of 5°C min^-1^ and maintained for 2 hours in the argon. Subsequently, the resulting black powder was stirred in HF (10 wt%) for 12 hours at room temperature to remove the template. Finally, the powder was treated with 1.0 M H_2_SO_4_ at 80 °C for 24 hours and then washed with deionized water until neutral. The obtained catalyst was vacuum dried at 60 °C overnight.

**Preparation of** **FeSNC**

First, 100.0 mg of FeTThP was dissolved in 100.00 ml of chloroform, and then 100.0 mg of hydrochloric acid-treated SBA-15 and 100.0 mg anhydrous ferric chloride were added to the solution. The mixture was kept under sonication and vacuum at room temperature for 1 hour and then stirred vigorously at 40 °C for 24 hours under Ar protection. Chloroform and FeCl_3_ were removed by rotary evaporation and washing with deionized water, respectively. Like the preparation process of FeNC, the obtained composite was subsequently subjected to pyrolysis, HF etching, and H_2_SO_4_ treatment.

**Preparation of FeNCS**

The synthesis process of FeNCS is similar to that of FeNC. The only difference is that 10.0 mg of thiourea was added during the synthesis of FeNCS.

1. Characterizations

All ^1^H NMR spectroscopy spectra were recorded on a Bruker Avance-300 MHz spectrometer at 295 K. All spectra were obtained from deuterated chloroform. The UV-Vis spectra were recorded using an Ocean Insight QE65Pro High-Sensitivity spectrometer. All solutions use DMF as a solvent. The X-ray diffraction (XRD) patterns were obtained by employing a Rigaku D/Max2550 instrument equipped with Cu Kα radiation (λ=1.5418 Å). The chemical properties of surface elements in materials were analyzed using X-ray photoelectron spectroscopy (XPS) with an Al Kα source on a Thermo Electron Corporation ESCALAB 250 spectrometer. Raman spectra were conducted using a Jobin Yvon/HORIBA LabRam ARAMIS Raman spectrometer with an excitation of 532 nm laser light. The morphology and element distribution of the catalysts were examined using scanning electron microscopy (SEM, JSM-6510) and transmission electron microscopy (TEM, Philips-FEI Tecnai G 2S-Twin F20) that were equipped with energy dispersive X-ray spectroscopy (EDX). The Nitrogen adsorption/desorption isotherms recorded on a Micromeritics ASAP 3-flex at 77 K. Brunauer-Emmett-Taller (BET) and Barrett-Joyner-Halenda (BJH) methods were used to determine the specific surface area and pore size distribution of catalysts, respectively. The concentrations of Fe present in the catalysts were determined through the use of inductively coupled plasma optical emission spectroscopy (ICP-OES) analysis, using the Perkin-Elmer Optima 3300 DV. Gas analysis during electrolysis was conducted using a Shimadzu GC-2014 gas chromatography (GC) system equipped with a thermal conductivity detector (TCD) and a flame ionization detector (FID).

Attenuated total reflectance surface-enhanced infrared spectroscopy (ATR-SEIRAS) was carried out on a Bruker vertex 80v machine with a mercury cadmium telluride (MCT) detector. Spectra were obtained at a 4 cm^-1^ spectral resolution with 32 scans. A CO_2_-saturated 0.5 M KHCO_3_ solution served as the electrolyte, with continuous CO_2_ supply during testing. Catalyst ink was uniformly applied to a germanium crystal coated with a gold film, serving as the working electrode. The reference electrode was Ag/AgCl, and the counter electrode was Pt wire. Background spectra were collected at open circuit potential. The potential ranged from -0.18 V *vs* RHE to -0.98 V *vs* RHE, with spectra collected every 100 mV.

1. Electrochemical Measurements

**Cyclic voltammetry (CV) in organic solvents.**

All electrochemical measurements were performed using a CHI660E workstation. A standard 3-electrode configuration was employed in the study, utilizing a freshly polished glassy carbon disk as the working electrode (WE), a platinum wire as the counter electrode (CE), and a silver/silver chloride reference electrode (RE). The preparation of a sample solution in an anhydrous DMF solvent involved the addition of 1 mM porphyrin and 0.1 M tetrabutylammonium hexafluorophosphate (TBAPF_6_) supporting electrolyte. Before measurement, the electrolyte was sparged with dry argon for 30 minutes. The cyclic voltammetry experiments were conducted with a scan rate of 100 mV s^-1^.

**CO_2_RR in the H-Cell.**

Disperse 5.0 mg of catalyst and 50 μL Nafion (5 wt%) into 450 μL ethanol/water (v : v = 4 : 5) mixture and sonicate for 30 minutes to form a uniform suspension. Subsequently, 50 μL of ink was drop-coated on carbon paper (Y30T, 1 × 1 cm^2^) to prepare a working electrode. All electrochemical measurements were conducted using a CHI 660E analyzer. The H cell chambers were separated by Nafion-117. The working electrode, counter electrode, and reference electrode were catalyst-coated carbon paper, platinum foil, and Ag/AgCl (in saturated KCl electrolyte), respectively. A 0.5 M KHCO_3_ electrolyte was utilized, and pure CO_2_ was bubbled into the electrolyte for 30 minutes before the test.

**Zn-CO_2_ battery measurements.**

Zn-CO_2_ battery efficiency was assessed in a two-chamber system separated by Fumasep FBM-PK. The anode employed a 2 × 5 cm^2^ zinc sheet, while the cathode utilized a 1 × 1 cm^2^ hydrophobic carbon paper loaded with 0.5 mg cm^-2^ catalyst. The catholyte solution contained 0.5 M KHCO_3_, and the anolyte solution comprised 6 M KOH and 0.2 M Zn(AcO)_2_. Throughout the experiment, CO_2_ was continuously introduced into the catholyte at a flow rate of 20 sccm.

1. Methods

**Calculation of Faradic efficiency (FE).**

$${FE}_{g}=\frac{Q_{g}}{Q_{total}}\times100\%=\frac{2\times\frac{V_{g}}{V_{m}}\times F\times100\%}{Q_{total}}$$

Where V_g_ is the volume of CO or H_2_ above the cathode chamber (L). V_m_ is the molar volume of gases at 25 °C (24.5 L mol^-1^). 2 is the number of electrons transferred to produce a molecule of CO or H_2_. F is Faraday constant (96485 C mol^-1^). Q_total_ is the charge consumed to produce all products. (C).

**Calculation of Turnover frequency (TOF).**

The turnover frequency (h^-1^) for CO was calculated as follows:

$$TOF=\frac{j_{total}\times{FE}_{CO}\times A\times M_{Fe}\times t}{2F\times\omega_{Fe}\times m}$$

Where *j*_total_ is the total current density (A cm^-2^), FE_CO_ is the Faradic efficiency of CO, and ω_Fe_ is the mass fraction of iron on the catalyst (analyzed by ICP-OES). F is Faraday constant (96485 C mol^-1^). A is the electrode geometric area (1 cm^2^). M_Fe_ is the atomic mass of Fe (55.84 g mol^-1^). m is the mass of the catalyst coated on the working electrode (0.5 mg). t is the reaction time (1 h/3600 s).

1. DFT calculations

All calculations were implemented in Materials Studio with the DMol^3^ code. The Perdew–Burke–Ernzerhof (PBE) functional of the generalized gradient approximation (GGA) was used to calculate exchange-correlation energy. The double numerical plus polarization (DNP) was chosen during the geometry optimization. A 3 × 3 × 1 Monkhorst-Pack k,points grid was used for k-points sampling, and 517 eV plane-wave expansion was setup for energy cut-offs. The convergence tolerances of energy change, maximum force, and maximum displacement were set as 2 × 10^-5^ Ha, 0.004 Ha/Å, and 0.005 Å, respectively. A 4 × 4 × 1 super cell graphene with vacuum layer of 15 Å was used to simulate the catalyst surface.

In this work, the elementary steps on the catalysts were:

$$*+{CO}_{2}+H^{+}+e^{-}\to*COOH$$

$$*COOH+H^{+}+e^{-}\to*CO+H_{2}O$$

$$*CO\to*+CO$$

The Gibbs free energy change is defined as:

*ΔG = ΔE + ΔZPE – TΔS*

where ΔE is the reaction energy calculated based on DFT calculations, ΔZPE and ΔS are the zero-point energy difference and the entropy change between the products and reactants, respectively, and T is the temperature (298.15 K). The zero-point energies and entropies of the reaction species were determined based on the vibrational frequencies. Throughout these frequency calculations, the substrate atoms were rigidly constrained to prevent the introduction of any additional degrees of freedom by the catalyst into the reacting system. For non-adsorbed gas-phase molecules, the entropies of H_2_ (g), CO_2_ (g), and CO (g) at 1.0 atm are used, while the entropy of H_2_O (l) is calculated at 0.035 atm, corresponding to the vapor pressure of liquid water at 300 K.

1. Supplementary Figures and Tables.

**
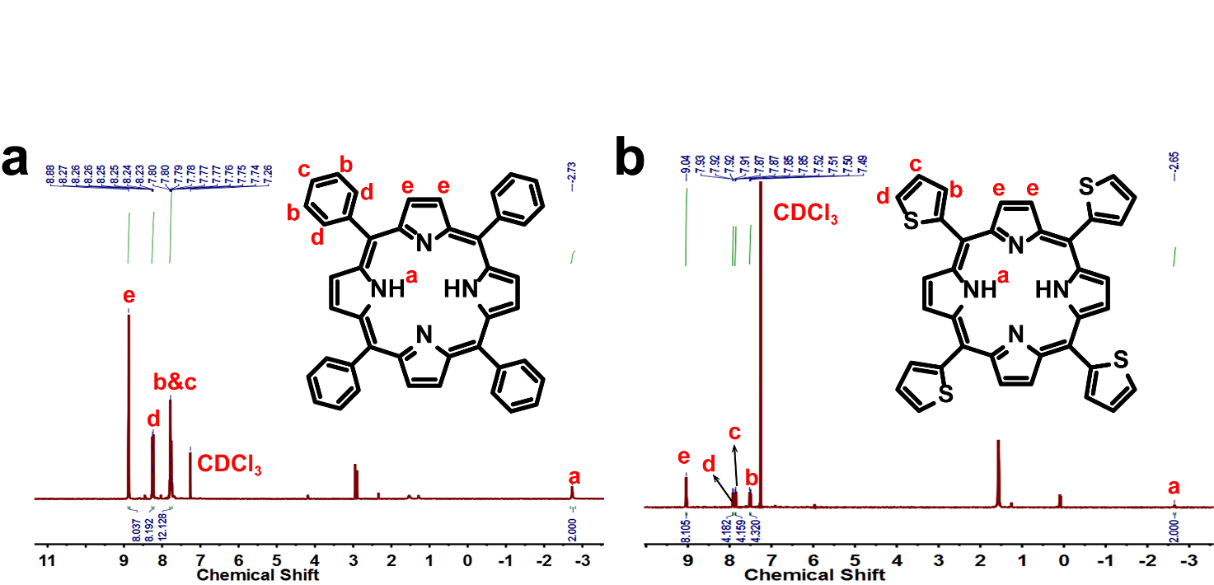
**

**Figure S1.** ^1^H NMR spectra of H_2_TPP and H_2_TThP.

**

**

**Figure S2.** The UV-visible absorption spectra of (a) H_2_TPP, (b) FeTPP, (c) H_2_TThP, and (d) FeTThP.





**Figure S3.** The mass spectrometry spectra of (a) H_2_TPP, (b) H_2_TThP, (c) FeTPP, and (d) FeTThP.





**Figure S4.** Cyclic voltammograms of (a) FeTPP, and (b) FeTThP in DMF with a scan rate of 100 mV s^-1^. All electrolytes contained ~1.0 mM catalyst and 0.1 M TBAPF_6_.





**Figure S5.** Fourier transformation EXAFS spectra of FeSNC and reference samples.





**Figure S6.** Fe K-edge EXAFS fitting curves of FeSNC in k space.





**Figure S7.** WT k^2^-weighted EXAFS contour plot of the (a) Fe foil, (b) FeO, (c) Fe_2_O_3_, (d) FePc, and (e) FeS_2_.





**Figure S8.** The S 1s spectra of FeSNC.


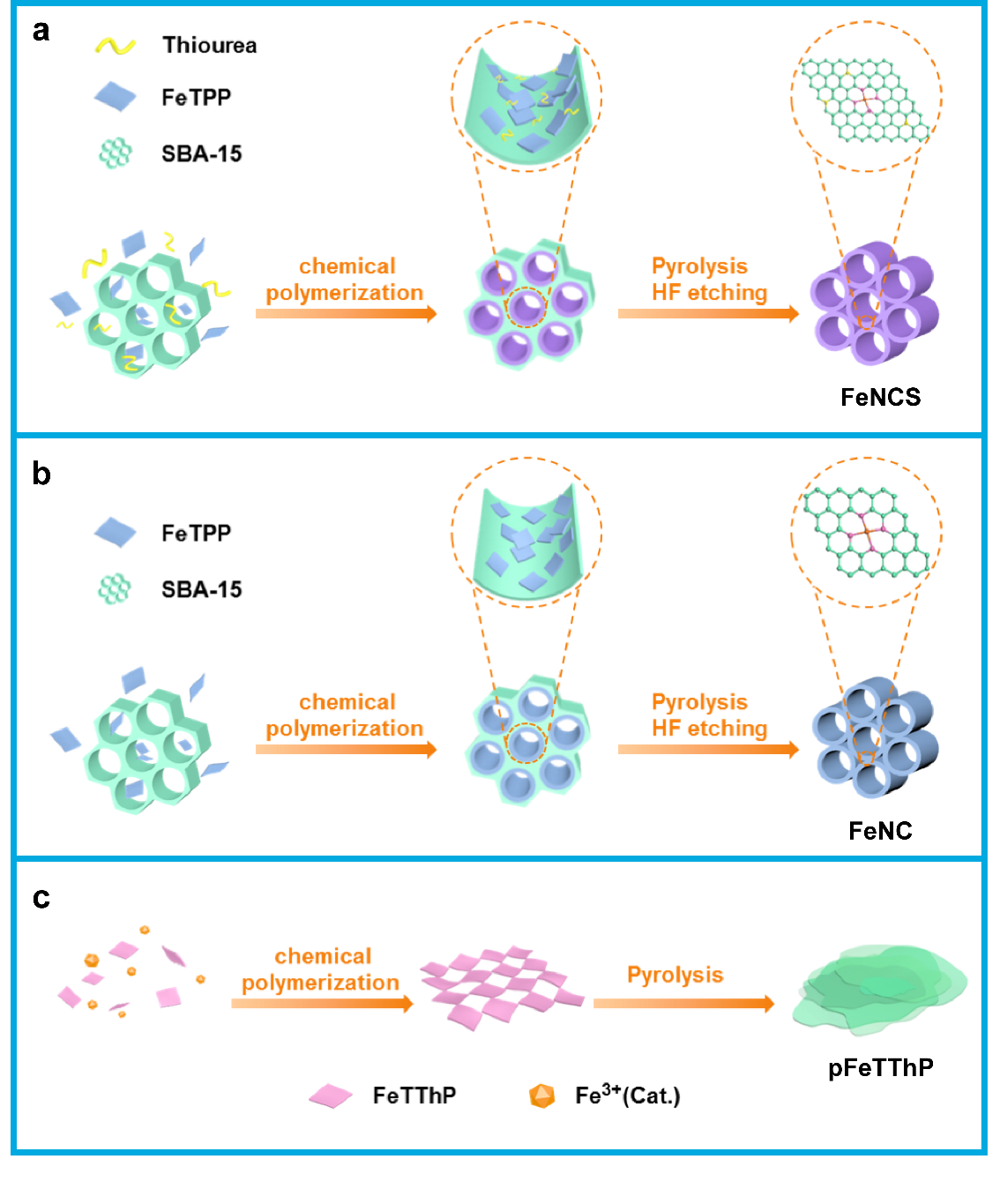


Figure S9. Schematic illustration of the synthesis of (a) FeNCS, (b) FeNC, and (c) pFeTThP.





**Figure S10.** SEM images of (a) FeNC, (b) FeNCS, and (c) pFeTThP.





**Figure S11.** TEM images of (a) FeNC, (b) FeNCS, and (c) pFeTThP.





**Figure S12.** Pore size distribution of SBA-15.





**Figure S13.** XRD patterns of FeNC-1100, FeNCS-1100, and FeSNC-1100.





**Figure S14.** Content of Fe derived from the ICP-OES analysis.





**Figure S15.** XPS spectra of as-prepared samples.





**Figure S16.** The Fe 2p spectra of FeNC, FeNCS, FeSNC, and pFeTThP.

**

**

**Figure S17.** The C 1s spectra of (a) FeNC, (b) FeNCS, (c) FeSNC, and (d) pFeTThp.





**Figure S18.** Raman spectra of FeNC, FeNCS, and FeSNC.





**Figure S19.** The N 1s spectra of (a) FeNC, (b) FeNCS, (c) FeSNC, and (d) pFeTThp.





**Figure S20.** The S 2p spectra of (a) FeNCS, and (b) pFeTThp.





**Figure S21.** i-t curves of (a) pFeTThp, (b) FeNC, (c) FeNCS, and (d) FeSNC.





**Figure S22.** Stability tests were performed in CO_2_-saturated 0.5 M KHCO_3_.





**Figure S23.** Raman specta for FeSNC-T.





**Figure S24.** Electrical conductance of FeSNC-T.

*

*

**Figure S25.** Pore size distribution of (a) FeSNC-900, (b) FeSNC-1000, and (c) FeSNC-1100.





**Figure S26.** Electrochemical surface area measurements were performed in Ar-saturated 0.5 M KHCO_3_.





**Figure S27.** Nyquist plots were performed in CO_2_-saturated 0.5 M KHCO_3_.





**Figure S28.** *In situ* ATR-SEIRAS spectroscopy of FeSNC at different applied potentials.





**Figure S29.** LSV curves in 0.5 M KHCO_3_ solution at with scan rate of 5 mV s^-1^.





**Figure S30.** Tafel plots in 0.5 M KHCO_3_ solution.


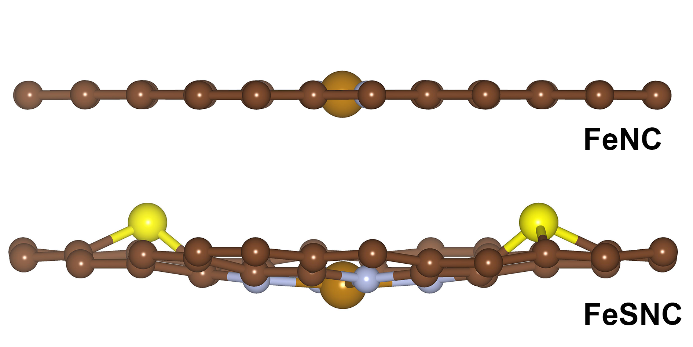


**Figure S31.** Side views of atomistic structures of FeNC (upper) and FeSNC (lower) catalysts. The brown, blue, golden, and yellow balls represent C, N, Fe, and S atoms, respectively.


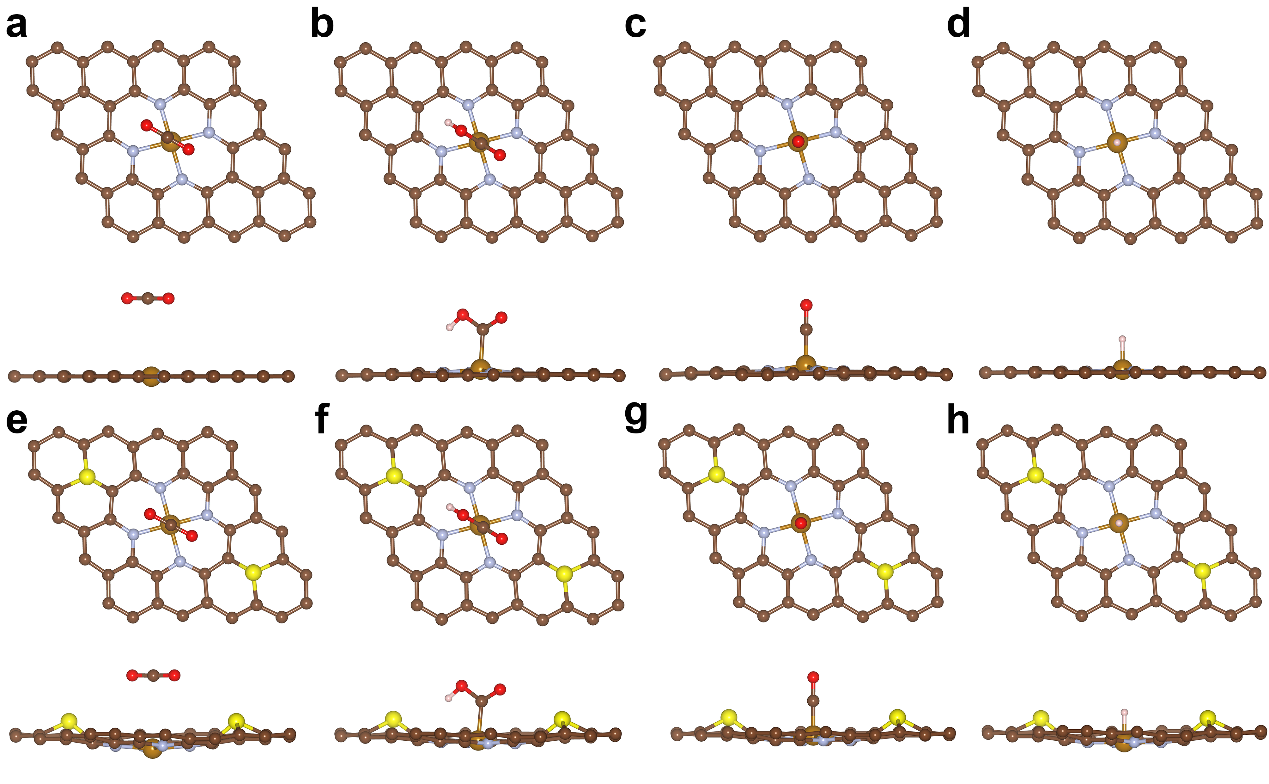


**Figure S32.** Top views and side views of FeNC (upper) and FeSNC (lower) catalysts after the (a, e) *CO_2_, (b, f) *COOH, (c, g) *CO, and (d, h) *H intermediates were adsorbed near the Fe atom. The brown, blue, golden, white, and yellow balls represent C, N, Fe, H, and S atoms, respectively.





**Figure S33.** Limiting potential difference between the CO_2_RR and HER (U_L_(CO_2_)-U_L_(H_2_)) on FeNC and FeSNC.

**Table S1.** EXAFS fitting parameters at the Fe K–edge for various samples

| Sample | Path | N | R（Å） | σ^2^（🞨10^-3^ Å^2^） | | E_0_（ev） | R-factor |
| --- | --- | --- | --- | --- | --- | --- | --- |
| Fe foil | Fe-Fe | 12 | 2.48±0.004 | 5.95±0.47 | -0.57±0.67 | | 0.0046 |
| FeSNC | Fe-N | 3.89(0.32) | 11.30(0.04) | 5.44(2.39) | -0.061(1.84) | | 0.014 |

*^a^CN*, coordination number; *^b^R*, the distance between absorber and backscatter atoms; *^c^σ*^2^, Debye-Waller factor to account for both thermal and structural disorders; *^d^ΔE*_0_, inner potential correction; *R* factor indicates the goodness of the fit. S_0_^2^ was fixed to 0.81 according to the experimental EXAFS fit of Fe foil by fixing CN as the known crystallographic value. A reasonable range of EXAFS fitting parameters: 0.600 < *Ѕ*_0_^2^ < 1.000; *CN >* 0; *σ*^2^ > 0 Å^2^; |Δ*E*_0_| < 15 eV; *R* factor < 0.02.

**Table S2.** The nitrogen contents of catalysts derived from XPS results.

|  | pFeTThP | FeNC | FeNCS | FeSNC |
| --- | --- | --- | --- | --- |
| S (at.%) | 2.31 | - | 0.89 | 1.19 |
| N (at.%) | 2.70 | 2.41 | 3.05 | 3.5 |
| Pyridnic-N (%) | 33.56 | 20.75 | 38.05 | 43.18 |
| Pyrrolic-N (%) | 24.37 | 20.78 | 24.47 | 27.19 |
| Graphitic-N (%) | 22.97 | 32.02 | 19.90 | 22.29 |
| Oxidized-N (%) | 19.10 | 26.44 | 17.57 | 7.34 |
| Fe (at.%) by XPS | 0.78 | 0.69 | 0.96 | 0.98 |
| Fe (wt.%) by ICP-OES | 0.06 | 0.20 | 0.17 | 0.13 |

**Table S3.** Physicochemical properties of SBA-15 and catalysts.

| Sample | BET surface area (m^2^g^-1^) | Pore volume (cm^3^g^-1^) |
| --- | --- | --- |
| SBA-15 | 727.70 | 1.54 |
| FeNC-1100 | 553.52 | 0.64 |
| FeNCS-1100 | 639. 66 | 0.68 |
| FeSNC-1100 | 605.52 | 0.73 |

**Table S4.** Physicochemical properties of FeSNC-T catalysts.

| Sample | Fe (wt%) by ICP-OES | Pore volume (cm^3^g^-1^) | BET surface area (m^2^g^-1^) |
| --- | --- | --- | --- |
| FeSNC-900 | 0.12 | 0.60 | 470.40 |
| FeSNC-1000 | 0.18 | 0.38 | 322.31 |
| FeSNC-1100 | 0.13 | 0.73 | 605.52 |

**Table S5.** Conductivity^#^ of FeSNC-T catalysts.

| Sample | Conductance (S) | Length (mm) | Conductivity (S/m) |
| --- | --- | --- | --- |
| FeSNC-900 | 0.05 | 0.250 | 0.16 |
| FeSNC-1000 | 0.11 | 0.277 | 0.39 |
| FeSNC-1100 | 0.56 | 0.485 | 3.46 |

^#^: The test is conducted using the tableting method with a disc diameter of d = 10 mm at room temperature.

**Table S6.** Performances comparison of our catalysts with other recently reported representative nickel-based catalysts for Zn-CO_2_ batteries

| **Catalysts** | **Maximum powder density (mW cm^-2^)** | **Cycle current density (mA cm^-2^)** | **Cycle time (h)** | **Reference** |
| --- | --- | --- | --- | --- |
| **FeSNC** | **1.19** | **0.5** | **53** | **This work** |
| Fe_1_NC/S_1_-1000 | 0.60 | 0.5 | 25 | ^[5]^ |
| Ni-N_3_-NCNFs | 1.10 | 2.0 | 45 | ^[6]^ |
| CA/N-Ni | 0.50 | 0.5 | 30 | ^[7]^ |
| Ni-N_3_-C | 1.1 | 2.0 | 100cycles | ^[8]^ |
| NiFe-DASC | 1.40 | 5.0 | 90 | ^[9]^ |
| Fe_1_−Ni_1_−N−C | - | 1.1 | 15 | ^[10]^ |
| Fe-N_5_/DPCF | 1.30 | 0.5 | 25 | ^[11]^ |
| FeN_4_Cl/NC-7.5 | 0.54 | 0.5 | 15 | ^[12]^ |
| Fe-SA/BNC | 1.18 | 1.0 | 27 | ^[13]^ |
| Fe-P@NCPs | 0.85 | 0.5 | 168 | ^[14]^ |

**Table S7.** The reaction free energy of the elementary reaction step of CO_2_ reduction and hydrogen evolution on the two catalysts.

|  | CO_2_ | *COOH | *CO | CO | *H | 1/2H_2_ |
| --- | --- | --- | --- | --- | --- | --- |
| FeNC | 0 | 1.09 | -0.32 | 0.75 | 0.46 | 0 |
| FeSNC | 0 | 0.93 | -0.15 | 0.75 | 0.53 | 0 |

**Table S8.** The limiting potential (U_L_) of CO_2_ reduction and hydrogen evolution on the two catalysts.

|  | U_L_(CO_2_) | U_L_(H_2_) | U_L_(CO_2_) - U_L_(H_2_) |
| --- | --- | --- | --- |
| FeNC | -1.09 | -0.46 | -0.63 |
| FeSNC | -0.93 | -0.53 | -0.40 |

1. References

[1] M. Enterría, F. Suárez-García, A. Martínez-Alonso, J. M. D. Tascón, *Microporous Mesoporous Mater.* **2012**, *151*, 390.

[2] J. D. B. Koenig, J. Willkomm, R. Roesler, W. E. Piers, G. C. Welch, *ACS Appl. Energy Mater.* **2019**, *2*, 4022.

[3] A. D. Adler, F. R. Longo, J. D. Finarelli, J. Goldmacher, J. Assour, L. Korsakoff, *J. Org. Chem.* **1967**, *32*, 476.

[4] Y. Zhou, F. Liu, H. Wu, B. Qu, L. Duan, *Asian J. Chem.* **2015**, *27*, 616.

[5] T. Wang, X. Sang, W. Zheng, B. Yang, S. Yao, C. Lei, Z. Li, Q. He, J. Lu, L. Lei, L. Dai, Y. Hou, *Adv. Mater.* **2020**, *32*, 2002430.

[6] W. Zheng, Y. Wang, L. Shuai, X. Wang, F. He, C. Lei, Z. Li, B. Yang, L. Lei, C. Yuan, M. Qiu, Y. Hou, X. Feng, *Adv. Funct. Mater.* **2021**, *31*, 2008146.

[7] Y. Zhang, X. Wang, S. Zheng, B. Yang, Z. Li, J. Lu, Q. Zhang, N. M. Adli, L. Lei, G. Wu, Y. Hou, *Adv. Funct. Mater.* **2021**, *31*, 2104377.

[8] Y. Zhang, L. Jiao, W. Yang, C. Xie, H. L. Jiang, *Angew. Chem. Int. Ed.* **2021**, *60*, 7607.

[9] Z. Zeng, L. Y. Gan, H. Bin Yang, X. Su, J. Gao, W. Liu, H. Matsumoto, J. Gong, J. Zhang, W. Cai, Z. Zhang, Y. Yan, B. Liu, P. Chen, *Nat. Commun.* **2021**, *12*, 4088.

[10] L. Jiao, J. Zhu, Y. Zhang, W. Yang, S. Zhou, A. Li, C. Xie, X. Zheng, W. Zhou, S.-H. Yu, H.-L. Jiang, *J. Am. Chem. Soc.* **2021**, *143*, 19417.

[11] Z. Li, J. Jiang, X. Liu, Z. Zhu, J. Wang, Q. He, Q. Kong, X. Niu, J. S. Chen, J. Wang, R. Wu, *Small* **2022**, *18*, 2203495.

[12] Z. Li, R. Wu, S. Xiao, Y. Yang, L. Lai, J. S. Chen, Y. Chen, *Chem. Eng. J.* **2022**, *430*.

[13] S. Liu, M. Jin, J. Sun, Y. Qin, S. Gao, Y. Chen, S. Zhang, J. Luo, X. Liu, *Chem. Eng. J.* **2022**, *437*, 135294.

[14] S. Liu, L. Wang, H. Yang, S. Gao, Y. Liu, S. Zhang, Y. Chen, X. Liu, J. Luo, *Small* **2022**, *18*, 2104965.
